# About prestretch in homogenized constrained mixture models simulating growth and remodeling in patient-specific aortic geometries

**DOI:** 10.1007/s10237-021-01544-3

**Published:** 2022-01-24

**Authors:** Joan D. Laubrie, S. Jamaleddin Mousavi, Stéphane Avril

**Affiliations:** 1 Mines Saint-Étienne, University of Lyon, University of Jean Monnet, INSERM, U 1059 Sainbiose, 42023 Saint-Étienne, France; 2 Institute for Mechanics of Materials and Structures, TU Wien-Vienna University of Technology, 1040 Vienna, Austria

**Keywords:** Arterial growth and remodeling, Homogenized constrained mixture model, Layer-specific behavior, patient-specific, Prestretch, Finite element method

## Abstract

Evolution of mechanical and structural properties in the Ascending Thoracic Aorta (ATA) is the results of complex mechanobiological processes. In this work, we address some numerical challenges in order to elaborate computational models of these processes. For that, we extend the state of the art of homogenized constrained mixture (hCM) models. In these models, prestretches are assigned to the mixed constituents in order to ensure local mechanical equilibrium macroscopically, and to maintain a homeostatic level of tension in collagen fibers microscopically. Although the initial prestretches were assumed as homogeneous in idealized straight tubes, more elaborate prestretch distributions need to be considered for curved geometrical models such as patient-specific ATA. Therefore, we introduce prestretches having a three-dimensional gradient across the ATA geometry in the homeostatic reference state. We test different schemes with the objective to ensure stable growth and remodeling (G&R) simulations on patient-specific curved vessels. In these simulations, aneurysm progression is triggered by tissue changes in the constituents such as mass degradation of intramural elastin. The results show that the initial prestretches are not only critical for the stability of numerical simulations, but they also affect the G&R response. Eventually, we submit that initial conditions required for G&R simulations need to be identified regionally for ensuring realistic patient-specific predictions of aneurysm progression.

**Supplementary Information:**

The online version contains supplementary material available at 10.1007/s10237-021-01544-3.

## Introduction

The Ascending Thoracic Aorta (ATA) plays an essential role for the function of the cardiovascular system thanks to the Windkessel effect (Humphrey 2002). Similarly to other biological tissues, the ATA continuously adapts its structure and shape to accommodate aging effects (O’Rourke et al. 2008) and possible physiopathological changes, which in the long-term can unfortunately lead to diseases such as aneurysms (Hiratzka et al. 2010).

Such structural changes can be computationally predicted by Growth and Remodeling (G&R) models. These models date back from the mid-1990s when Rodriguez et al. (1994) introduced the Kinematics Growth (KG) model, which is based on a multiplicative decomposition of the deformation gradient into elastic and inelastic contributions. Some years after, Humphrey and Rajagopal (2002) proposed the Constrained Mixture (CM) model, which is based on the assumption that soft tissues are composite materials. In arteries, the main constituents of this composite material are elastin, collagen and smooth muscle cells (SMCs). Based on CM models, G&R explicitly simulates continuous mass deposition/removal of each constituent. Due to the high implementation efforts and computation resources required by CM models, some authors proposed hybrid models such as the Evolving Recruitment Stretch model (Watton et al. 2004; Eriksson et al. 2014), the Homogenized Constrained Mixture (hCM) model (Cyron et al. 2014; Braeu et al. 2017; Mousavi et al. 2019) or the rate-independent pseudoelastic framework (Latorre and Humphrey 2018, 2020).

Generally, geometries of organs and tissues that are considered to set-up numerical models are acquired in vivo and consequently the reference configuration cannot be assumed as stress-free (Bellini et al. 2014). A prestretch, i.e. a stretch that exists in the reference configuration of the body of interest (Maas et al. 2016), needs to be introduced. As most G&R computational models rely on the paradigm of tensional homeostasis, which states that a biological system produces and removes mass to reach a target stress metrics (Cyron et al. 2014; Cyron and Humphrey 2016), the prestretch can contribute significantly to the G&R response of the system. Therefore, inclusion of prestretch is needed in G&R computational models of biological tissues to obtain reasonable predictions of tissue mechanics. In CM models, the prestretch is specific to each constituent (Bellini et al. 2014; Mousavi and Avril 2017).

Regional variations of prestretch in G&R models have been rarely investigated as most of previous work about vascular mechanobiology was achieved in quasi-straight patient-specific tubes (Zeinali-Davarani et al. 2011b) or in idealized straight arteries representing either the abdominal aorta (Figueroa et al. 2009; Wilson et al. 2012; Valentín et al. 2013; Horvat et al. 2019; Laubrie et al. 2019) or brain arteries (Baek et al. 2006). Nevertheless, Alford and Taber (2008) found regionally varying prestretches in a torus—representing an idealized aortic arch—after G&R simulations. More recently, Mousavi et al. (2019) performed G&R simulations in a torus again, and, for the first time ever, in patient-specific ATA geometries. They assigned spatially uniform prestretches for each constituent, named deposition stretches. As uniform prestretches do not guarantee global equilibrium of the ATA structure, Mousavi et al. (2019) resorted to radial rollers, which were used to assign a supplemental kinematic constraint to each point of the model. Although their G&R simulations showed very realistic predictions of ATA aneurysm progression, the radial rollers represent a limitation as they do not represent a physical reality.

In the current work, we investigate G&R models for patient-specific ATA. As ATA is a curved artery, this makes the task of assigning initial prestretches quite challenging. Previous work on this topic is rather scarce. Therefore, our objective is to find the prestretch conditions permitting G&R simulations in ATA curved geometries without resorting to the radial rollers. In order to accomplish this, we propose to use non-uniform prestretches in order to define the initial homeostatic state within the hCM model.

The manuscript is organized as follows: in Sect. 2, we give details about the G&R background, estimation of the prestretch gradient and the numerical implementation of our approach with its respective verification. At the end of Sect. 2, in Sect. 2.6, we describe the numerical tests used to demonstrate the robustness of our G&R implementation. After showing the obtained results in Sect. 3, we eventually propose, in Sect. 4, a discussion about the significance of prestretches in G&R patient-specific models.

## Materials and methods

###  Background on homogenized constrained mixture mechanics

**Fig. 1:**
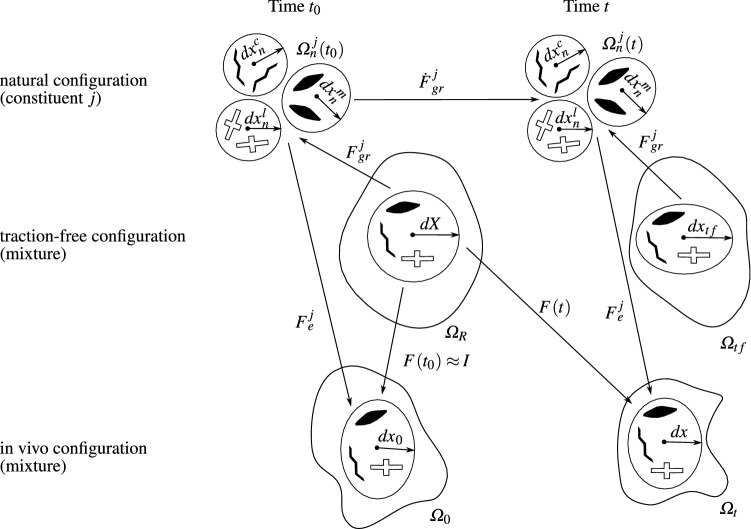
Schematic of the hCM model, showing the different configurations. The reference configuration $\Omega _R$ is reconstructed from the actual in vivo geometry of the artery. The configuration $\Omega _0$ is obtained by applying the initial boundary conditions and by assigning initial prestretches $[\pmb {F}_{\text {gr}}^j]^{-1}$ to each constituent of $\Omega _R$. $\Omega _R$ and $\Omega _0$ should be the same as there should be equilibrium between the effects of the initial boundary conditions and the effects of the initial prestretches in the reference configuration. However, both are represented separately in the figure as the initial prestretches providing this equilibrium are found iteratively in our approach. The fictitious traction-free configuration $\Omega _{tf}$ is defined as a fictitious configuration at time *t*, without the effects of boundary conditions and of prestretches. The current configuration $\Omega _t$ is obtained after equilibrium between the effects of the current boundary conditions and the effects of the updated prestretches obtained after growth and remodeling. The neighborhood ${\text {d}}\pmb {X}$ of an arbitrary point in $\Omega _R$ is related to $\Omega _t$ by the transformation ${\text {d}}\pmb {x}=\pmb {F}{\text {d}}\pmb {X}$. At time zero, $\Omega _t=\Omega _0$ and ${\text {d}}\pmb {x}_0=\pmb {F}{\text {d}}\pmb {X}$. Similarly, the relationship between $\Omega _t$ and the natural configuration is ${\text {d}}\pmb {x}=\pmb {F}^j_ed\pmb {x}^j_n$, and the natural configuration and $\Omega _R$ are related by the inelastic deformation ${\text {d}}\pmb {x}^j_n=\pmb {F}^j_{\text {gr}}{\text {d}}\pmb {X}$ where the inelastic deformation evolves with time. The natural configurations $\Omega _n^i(t)$ can only be defined locally but are not compatible

Let us consider an unloaded body $\Omega _R$, made of a mixture of several constituents. These constituents are smooth muscle cells (*m*), extracellular matrix containing collagen fiber families ($c_i$) and the remaining matrix (*l*) composed of elastin and of a ground substance including glycosaminoglycans (Caulk et al. 2018; Humphrey et al. 2015). Each constituent of the mixture is denoted with letter *j* as in Fig. 1. When the mixture undergoes a deformation from the reference configuration ($\Omega _R$) to a deformed configuration ($\Omega _t$), all the constituents of the mixture have the same deformation gradient $\pmb {F}$ at a given position. In the hCM model, this deformation gradient is split into an inelastic and an elastic part, both being specific to each constituent. Then, it may be written at any time *t*,

1 $$\begin{aligned} \pmb {F}(\pmb {X},t) = \pmb {F}^j_e(\pmb {X},t)\pmb {F}^j_{\text {gr}}(\pmb {X},t), \end{aligned}$$

where $\pmb {F}^j_e$ is the elastic part of the deformation gradient in the *j*th constituent and $\pmb {F}^j_{\text {gr}}$ is the inelastic part of the deformation gradient in the *j*th constituent. The stresses, related to the elastic part, satisfy equilibrium equations, whereas the inelastic part relates to the permanent deformations resulting from G&R (Braeu et al. 2017; Cyron et al. 2016; Rodriguez et al. 1994) (for more details, see the supplemental materials, sections A and C).

The constituents are assumed to be hyperelastic. The strain energy density function *W* (per unit reference volume) of the mixture of *n* constituents is defined as

2 $$\begin{aligned} W(\pmb {C}) = \sum _j^n\varrho ^j_R\Psi ^j(\pmb {C}^j_e)= \sum _j^n\varrho ^j_R\Psi ^j\left( {\pmb {F}^j_{\text {gr}}}^{-T} \pmb {C}~{\pmb {F}^j_{\text {gr}}}^{-1}\right) , \end{aligned}$$

where $\pmb {C}=\pmb {F}^T\pmb {F}$ is the right Cauchy–Green stretch tensor, $\pmb {C}^j_e={\pmb {F}^j_e}^T \pmb {F}^j_e$ is the elastic part of the right Cauchy–Green stretch tensor, $\Psi ^j$ is the *j*th constituent strain energy function (per unit reference mass), which depends only on $\pmb {C}^j_e$, $\varrho ^j_R$ is the mass density (per unit reference volume), in the *j*th constituent. Complete details about of the hyperelastic constitutive models can be found in supplemental materials, section B. Then we can derive the stress and tangent stiffness tensors, which are needed in our finite-element implementation, and which are written, respectively, as

3 $$\begin{aligned} \pmb {S} = \sum _j^n\varrho ^j_R\frac{\partial \Psi ^j(\pmb {C}^j_e)}{\partial \pmb {C}} \text { and } \mathbb {C} = \sum _j^n\varrho ^j_R\frac{\partial ^2\Psi ^j(\pmb {C}^j_e)}{\partial \pmb {C}^2}, \end{aligned}$$

where $\pmb {S}$ is the second Piola–Kirchhoff and $\mathbb {C}$ is the elasticity tensor for the material stiffness.

###  Growth and remodeling based on homogenized constrained mixture models

G&R considers temporal evolutions related to the mass changes of the different constituents of the mixture. The idea of the hCM models is to use temporal homogenization in order to pool all the sequential changes within one single inelastic deformation gradient for each constituent (Fig. 1). As G&R is a stress mediated process, we assumed that mass can be added or removed to minimize deviations between a convenient stress equivalent in the current state and a reference homeostatic stress value. This requires a continuous update of $\pmb {F}^j_{\text {gr}}(\pmb {X},t)$ in order to account for the changes of tissue microstructure, which are referred as remodeling, and which result from this continuous mass deposition and removal. Indeed, due to the ongoing mass deposition and removal, the natural configuration of each constituent continuously changes during G&R (Fig. 1), even when there is a balance between mass deposition and removal ($\dot{\varrho }^j_R=0$). More details about the numerical implementation of G&R are given in supplemental materials, section C.

### Assigning non uniform initial prestretches

#### General statements about initial prestretches

As shown in Fig. 1, we can define the following configurations:

- $\Omega _R$ is the reference configuration. $\Omega _R$ is reconstructed from the actual in vivo geometry of the artery.
- $\Omega _0$ is the configuration obtained by applying the initial boundary conditions and by assigning initial prestretches to each constituent of $\Omega _R$. $\Omega _R$ and $\Omega _0$ should be the same as there should be equilibrium between the effects of the initial boundary conditions and the effects of the initial prestretches in the reference configuration. Therefore, all points of the body are at position $\pmb {X}$ at time $t_0$ and we consider small neighborhoods as ${\text {d}}\pmb {X}$ in this configuration.
- $\Omega _t$ is the spatial configuration of the body, defined with positions $\pmb {x}(\pmb {X},t)$ deformed from the reference coordinates under the effects of the current boundary conditions and the current prestretches. The deformed neighborhood is ${\text {d}}\pmb {x}(\pmb {X},t)=\pmb {F}(\pmb {X},t){\text {d}}\pmb {X}$
- $\Omega _n^j(t)$ are the local natural stress-free configurations of the *j*th constituents of the mixture, defined with positions $\pmb {x}_n^j(\pmb {X},t)$ such as the local and incompatible neighborhoods of each constituent are given by ${\text {d}}\pmb {x}_n^j(\pmb {X},t)=\pmb {F}^j_{\text {gr}}(\pmb {X},t){\text {d}}\pmb {X}$. Those local neighborhoods can be re-assembled in the spatial configuration by a new deformation such as ${\text {d}}\pmb {x}(\pmb {X},t)=\pmb {F}^j_{e}(\pmb {X},t){\text {d}}\pmb {x}_n^j(t)$, where the resulting relationship $\pmb {F}^j_{e}=\pmb {F}[\pmb {F}^j_{\text {gr}}]^{-1}$ may be seen as a prestretch and is sometimes called deposition stretch, as collagen fibers or smooth muscle cells are naturally under tension when they are deposited at homeostasis (Bellini et al. 2014).

A major characteristic of hCM models is that, at a chosen reference time $t_0$, ${\pmb {F}^j_{\text {gr}}(\pmb {X},t_0)}\ne \pmb {I}$ for each constituent. Assuming homeostatic conditions at $t_0$, the prestress tensor of collagen fibers and of smooth muscle cells satisfy $\pmb {\sigma }_h^j(\pmb {X},t_0) = \sigma ^j_{h}~\pmb {a}^j_0\otimes \pmb {a}^j_0$, where $\pmb {a}^j_0$ is the vector indicating the direction of the main axis of collagen fibers or smooth muscle cells at $t_0$ (Cyron et al. 2016). The elastic models for collagen and SMCs are presented in supplemental materials, section B.

Then, the prestretch tensors of collagen fibers and of smooth muscle cells, ${\pmb {F}^{c_i}_{e}}$ and ${\pmb {F}^m_{e}}$, respectively, satisfy

4 $$\begin{aligned} \pmb {F}^j_{e}(\pmb {X},t_0) = \lambda ^j_{h}\pmb {a}^j_0\otimes \pmb {a}^j_0 + \frac{1}{\sqrt{\lambda ^j_h}}(\pmb {I}-\pmb {a}^j_0\otimes \pmb {a}^j_0), \end{aligned}$$

where $\lambda ^j_{h}$ is the prestretch of fibre *j* at homeostasis. This deposition stretch can be determined experimentally (Bellini et al. 2014).

The prestretch of the extracellular matrix is partially defined with the collagen prestretch. However, the remaining matrix (composed mainly of elastin but also of a ground substance comprising glycosaminoglycans Caulk et al. 2018; Humphrey et al. 2015), which is further denoted with letter *l*, should also be assigned a prestretch tensor $\pmb {F}^l_{e}$. As Neo-Hookean (supplemental materials, section B), the Cauchy stress of this constituent should satisfy

5 $$\begin{aligned} \pmb {\sigma }^l(\pmb {X},t_0) = \frac{\rho ^l_R}{J}\mu ^l \left( {J^l_e}\right) ^{-2/3} \left( \bar{\pmb {b}}^l_e-\frac{1}{3}\text {tr}\left( \bar{\pmb {b}}^l_e\right) \pmb {I}\right) + \frac{\rho ^l_R}{J}\kappa ^l J^l_e\left( J^l_e -1\right) \pmb {I} , \end{aligned}$$

where $\bar{\pmb {b}}^l_{e}=\bar{\pmb {F}}^l_{e}\left[ \bar{\pmb {F}}^l_{e}\right] ^T$ is the modified left Cauchy–Green stretch tensor and $J^l_e=\det (\pmb {F}^l_{e})$. $\bar{\pmb {F}}^l_{e}$ is the isochoric elastic deformation, its determinant is $\text {det}(\bar{\pmb {F}}^l_{e})=1$ and it is defined as $\bar{\pmb {F}}^l_{e}={J^l_e}^{-1/3}\pmb {F}^l_{e}$.

Unlike collagen and SMCs, this constituent is not bound to satisfy homeostatic conditions. However, $\pmb {F}^l_{e}$ should ensure the mechanical equilibrium of the mixture (supplemental A) when an external force $\pmb {f}$ is applied on the body, which may be written such as

6 $$\begin{aligned} \text {div} \left( \pmb {\sigma }^l +\sum ^{m,c_i}_j \sigma ^j_{h}~\pmb {a}^j_0\otimes \pmb {a}^j_0 \right) +\pmb {f}=0. \end{aligned}$$

In order to perfectly define the initial conditions, we have to solve Eq. 6 and find $\pmb {F}_e^l(t_0)$ (prestretch of the matrix, surrounding collagen fibers and SMCs) such as,

7 $$\begin{aligned} {\text {d}}\pmb {x}_0 = \pmb {F}^l_{e}{\text {d}}\pmb {x}_n^l={\text {d}}\pmb {X}. \end{aligned}$$

The resolution of this problem is only possible if we know the tractions applied on the whole boundary of $\Omega _0$. When $\Omega _0$ is a segment of artery in vivo, these tractions are usually unknown at both cross-sectional ends. Indeed if we keep the ends of the segment constrained and free from the external loads (pressure for instance), the obtained configuration will not be stress-free and there will be elastic deformations (cf. Eq. (1) in Cyron et al. (2016)). We can also imagine to split the body into several small parts, where each part has just one constituent, but their assembly is not necessarily geometrically compatible. As a consequence, it is not possible to use $\pmb {F}^l_{e}=\partial \pmb {x}_0/\partial \pmb {x}_n^l$ as a valid definition for the prestretch (Bonet and RDWood 2008).

#### Assigning $\pmb {F}^l_{e}(\pmb {X},t_0)$ in a perfectly cylindrical straight tube

**Fig. 2:**
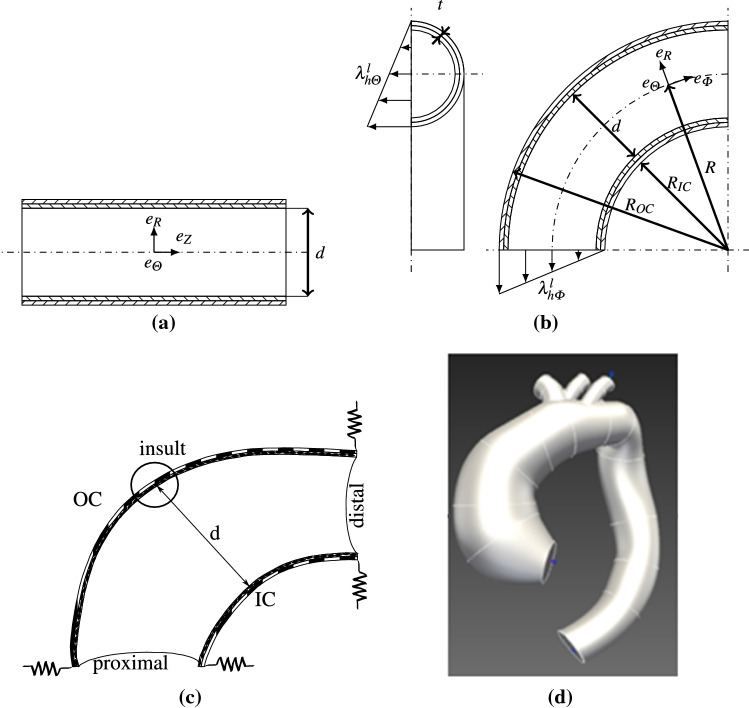
**a** Lateral view of a cylinder with its diameter *d* and the cylindrical system ($\pmb {e}_Z,\pmb {e}_R,\pmb {e}_{\Theta }$) with $\pmb {e}_{\Theta }$ perpendicular to the sheet. **b** Lateral and cross-sectional views of the idealized toric ATA model, where the luminal diameter is $d=R_{OC}-R_{IC}$, the arch radius is *R* (middle curvature) and the total wall thickness is *t*. IC = inner curvature and OC = outer curvature of the arch; a linear gradient is assigned for the axial ($\lambda _{h\Phi }^l$) and circumferential ($\lambda _{h\Theta }^l$) prestretch of elastin in the reference homeostatic state. The torus is represented with the spherical coordinate system ($\pmb {e}_{\Phi },\pmb {e}_R,\pmb {e}_{\Theta }$) with $\pmb {e}_{\Theta }$ perpendicular to the sheet. **c** Schematic of the boundary conditions, with springs at the proximal ($k_{pro}$) and at the distal ($k_{dis}$) ends; the circle (with radius $L_{\text {dam}}$) indicates the insult zone where a localized degradation of elastin is applied; the diameter *d* and the thickness along the same line are used to assess the initial distortions and displacements during the simulations. **d** Reconstructed geometry of the patient-specific aorta from the CT scan. In **a** and **b**, the media is filled with north west lines and the adventitia with north east lines, in **c** the media is filled with dots and the adventitia with vertical black thick lines

The problem can be easily overcome when $\Omega _{R}$ is a perfectly cylindrical straight tube. It can be assumed that the prestretch is a diagonal tensor within the cylindrical ($\pmb {X}=X_1\pmb {e}_Z+X_2\pmb {e}_R+X_3\pmb {e}_{\Theta }$, Fig. 2) reference frame, such as,

8 $$\begin{aligned} \pmb {F}^l_e(\pmb {X},t_0) = diag\left( \lambda ^l_{hZ}, \lambda ^l_{hR}, \lambda ^l_{h\Theta }\right) , \end{aligned}$$

where $\{\lambda ^l_{hZ},\lambda ^l_{hR},\lambda ^l_{h\Theta }\}$ are the elastic prestretches in the radial, circumferential and longitudinal direction, respectively.

Then, $\lambda ^l_{hZ}(\pmb {X},t_0)$ is fixed to an arbitrary value, which is equal to the supposed in vivo axial stretch of the artery, denoted $\lambda ^\text {iv}$ (known averagely for the human aorta Hornỳ et al. 2017) and assumed to be uniform in all directions. Eventually, $\lambda ^l_{h\Theta }$ and $\lambda ^l_{hR}$, which are assumed to be uniform across the cylinder, are directly obtained by solving Eq. 6 in the radial and tangential directions and by satisfying the incompressibility condition.

#### Assigning $\pmb {F}^l_{e}(\pmb {X},t_0)$ in a perfectly toric tube

Things become more complex for non-cylindrical shapes. We found that, when $\Omega _{R}$ is a perfectly toric tube (torus slice), compatibility equations could still be satisfied if we assumed that $\pmb {F}^l_e(\pmb {X},t_0)$ is a diagonal tensor within the local toric ($\pmb {X}=X_1\pmb {e}_{\Phi }+X_2\pmb {e}_R+X_3\pmb {e}_{\Theta }$) reference frame (where $\pmb {e}_\Theta$ refers to the poloidal or circumferential direction, and $\pmb {e}_\Phi$ refers to the toroidal or longitudinal direction), Fig. 2. However, it is necessary to consider regional variations of $\lambda ^l_{h\Phi }(\pmb {X},t_0)$, $\lambda ^l_{h\Theta }(\pmb {X},t_0)$ and $\lambda ^l_{hR}(\pmb {X},t_0)$. For that, we assumed $\lambda ^l_{h\Phi }(\pmb {X},t_0)$ only varying along the radial direction from the inner curvature to the outer curvature (Fig. 2) such as

9 $$\begin{aligned} \lambda ^l_{h\Phi }(R,t_0)=\lambda ^l_{h\Phi -IC}+(\lambda ^l_{h\Phi -OC}-\lambda ^l_{h\Phi -IC})\frac{R-R_{IC}}{d}, \end{aligned}$$

where *d* is the diameter of the tube and $R\in [R_{IC},R_{OC}]$. Additionally, the maximum $\lambda ^l_{h\Phi }$ is set at the outer curvature ($\lambda ^l_{h\Phi -OC}$) and is equal to the supposed in vivo axial stretch $\lambda ^\text {iv}$, while the minimum stretch is updated iteratively at the inner curvature ($\lambda ^l_{h\Phi -IC}$).

Therefore the circumferential prestretch, $\lambda ^l_{h\Theta }(R,t_0)$, satisfies a similar expression and the radial prestretch is assigned to satisfy the local incompressibility condition, yielding

10 $$\begin{aligned} \lambda ^l_{h\Theta }(R,t_0)=\lambda ^l_{h\Theta -IC}+(\lambda ^l_{h\Theta -OC}-\lambda ^l_{h\Theta -IC})\frac{R-R_{IC}}{d}, \end{aligned}$$

and

11 $$\begin{aligned} \lambda ^l_{hR}(R,t_0)=(\lambda ^l_{h\Theta } \lambda ^l_{h\Phi })^{-1}. \end{aligned}$$

Then, the two parameters $\lambda ^l_{h\Theta -IC}$ and $\lambda ^l_{h\Theta -OC}$ are found by solving Eq. 6 projected along the radial direction of the torus.

#### Assigning $\pmb {F}^l_{e}(\pmb {X},t_0)$ in a patient-specific aortic arch

**Fig. 3:**
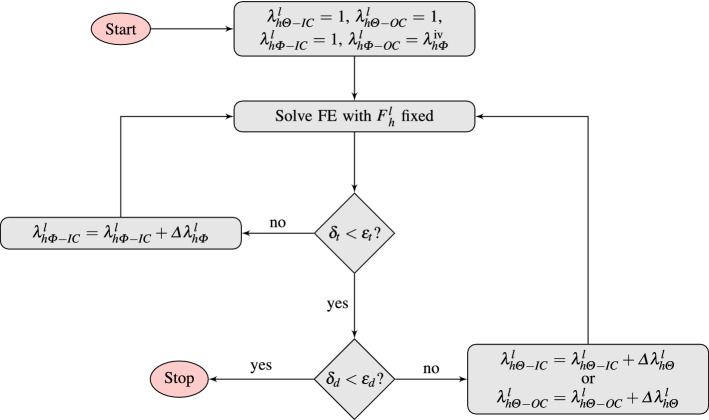
Flowchart for the homeostatic prestretch algorithm, showing how the prestretch gradient is found iteratively by solving forward FE problems successively. In the forward FE model, the prestretch gradient is held constant. After each forward analysis, the axial prestretch gradient ($\nabla \lambda ^l_{h\Phi }$) is updated if the thickness distortion ($\delta _t$) is larger than the thickness tolerance ($\epsilon _t$), or the circumferential prestretch gradient ($\nabla \lambda ^l_{h\Theta }$) is updated if the diameter distortion ($\delta _d$) is larger than the diameter tolerance ($\epsilon _d$) (Maas et al. 2016)

When $\Omega _{R}$ is not a perfectly toric tube, compatibility equations are not satisfied anymore if we assume that $\pmb {F}^l_e(\pmb {X},t_0)$ is a diagonal tensor. However, the torus is still a first approximation of the aortic arch. Therefore, for patient-specific geometries, we developed an iterative approach starting from the solution of Eq. 9, and thereafter updating it iteratively to address the distortions induced by the incompatibility of the assigned prestretch. This consisted in updating the axial prestretch at the inner curvature ($\lambda ^l_{h\Phi -IC}$), improving its values iteratively by reducing the thickness distortion (under the tolerance $\epsilon _t$=3%), and then in updating the circumferential prestretch at the outer and inner curvatures ($\lambda ^l_{h\Theta -OC}$ and $\lambda ^l_{h\Theta -IC}$, respectively) reducing the distortion of the diameter (under $\epsilon _d$=6%), Fig. 3.

#### Time evolutions of $\pmb {F}^j_{\text {gr}}(\pmb {X},t_0)$

The prestretch $\pmb {F}^j_{e}(\pmb {X},t_0)$ at $t_0$ equals the inelastic deformation gradient $\pmb {F}^j_{\text {gr}}(\pmb {X},t_0)$ of the constituents. This inelastic deformation gradient relates the natural stress-free configuration with an hypothetical traction-free configuration, which at $t_0$ is the reference configuration. During G&R the natural stress-free configuration continuously evolves due to the mass turnover and structure changes, see supplemental materials, section C. Therefore, the inelastic deformation is continuously changing according to

12 $$\begin{aligned} \pmb {F}^j_{\text {gr}}(t)=\pmb {F}^j_{\text {gr}}(t_0)+\int ^{t}_{t_0}\dot{\pmb {F}}^j_{\text {gr}}(\tau )d\tau . \end{aligned}$$

### Numerical implementation

The hCM model of G&R was implemented in an in-house research Finite-Element (FE) code based on Florence (Poya et al. 2017, 2018) (written in Python/C++). We implemented new routines for the code and further modified existing Florence routines. At the core, a forward Euler (explicit) time integration scheme was created for the hCM, according to the formulation presented in supplemental materials, section C. Another new routine was developed to assign an assembly of multiple mixture materials in one body, as for instance a bi-layer arterial wall. We also developed routines to assign Robin elastic boundary conditions at both ends of the tube, as explained in supplemental materials, section A. The meshes were generated using GMSH (Geuzaine and Remacle 2009).

### Verification

A simulation was performed on a cylinder. An insult was applied, corresponding to the same elastin degradation as the one considered previously by other authors (Wilson et al. 2012; Cyron et al. 2016; Braeu et al. 2017), further written in Eq. 13. The purpose of this simulation was to verify our model, as reference results were previously published for such problem (Braeu et al. 2017). The geometry, load, mechanical properties, densities, initial prestrain and mass turnover are reported in Table 1. Different mass-gain parameters were tested ($k^{c_i}_{\sigma }=${0.05, 0.09, 0.11, 0.15}$/T^{c_i}$) as in the reference results. The mass-gain parameters of the G&R model are introduced in details in supplemental material section C. The mesh was hexahedral and composed of 2$\times$15$\times$60 elements (thickness$\times$circumferential$\times$length) in a quarter cylinder.

**Table 1:** Mechanical parameters used to verify our model against the results of Braeu et al. (2017) for the development of an aneurysm in an idealized cylindrical geometry

Symbol	Value	
*Geometry and load*		
Radius	*r*	10mm
Length	*l*	90mm
Thickness	*t*	1.41mm
Pressure	*p*	100mmHg
*Mechanical properties*		
Neo-Hookean	$\mu ^l$	72J/kg
Bulk-modulus	$\kappa ^l$	100 $\times$ 72J/kg
Fung-quadratic, collagen	$k_1^{c_i}$	568J/kg
	$k_2^{c_i}$	11.2
Passive, SMC	$k_1^m$	7.6J/kg
	$k_2^m$	11.4
Active, SMC	$\sigma _{actmax}$	54kPa
	$\lambda ^m_0$	0.8
	$\lambda ^m_{\max }$	1.4
	$\lambda _{act}$	1.0
*Density*		
Elastin	$\varrho _{R0}^l$	241.5kg/m$^3$
SMC	$\varrho _{R0}^m$	157.5kg/m$^3$
Collagen(0 and $\pi /2$)	$\varrho _{R0}^{c_1}=\varrho _{R0}^{c_4}$	65.1kg/m$^3$
Collagen($-\pi /4$ and $\pi /4$)	$\varrho _{R0}^{c_2}=\varrho _{R0}^{c_3}$	241.5kg/m$^3$
*Initial remodeling (prestrain)*		
Elastin longitudinal	$\lambda _{rz}^l(t=0)$	$1.25^{-1}$
Elastin circumferential	$\lambda _{r\theta }^l(t=0)$	$1.34^{-1}$
SMC	$\lambda _r^m(t=0)$	$1.1^{-1}$
Collagen	$\lambda _r^{c_i}(t=0)$	$1.062^{-1}$
*Turnover period*		
Collagen and SMC	$T^{c_i}=T^m$	101days
Elastin	$T^l$	101years

**Fig. 4:**
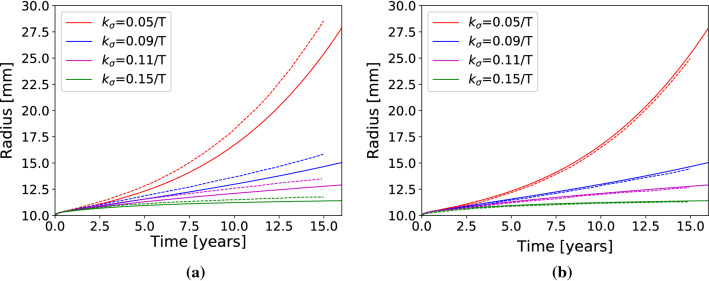
Evolution of the maximum radius for the cylinder benchmark case. 4 comparison between the radius predicted by our model (solid lines) with the three-dimensional model (dashed lines) of Braeu et al. (2017). 4 comparison between the radius predicted by our model (solid lines) with the membrane hCM model of Braeu et al. (2017)

The temporal evolutions of the maximum radius predicted by our model were in good agreement with the reference results of Braeu et al. (2017), as shown in Fig. 4. In Fig. 4, the comparison was performed against the three dimensional model of Braeu et al. (2017). The relative error is between 3% and 9%. In Fig. 4, the comparison was performed against the membrane model of Braeu et al. (2017). The relative error is even lower, ranging between 1% to 4%. The error is estimated 15 years after the original insult. In Table 2, we also compare the stress and normalized reference mass density of collagen, showing a good agreement between Braeu et al. (2017) and our model.

**Table 2:** Comparison of our results with results from (Braeu et al. 2017) for the development of an aneurysm in an idealized cylindrical geometry following an initial insult (localized elastin degradation)

		Literature (Braeu et al. 2017)		This work		Error	
		Minimum	Maximum	Minimum	Maximum	Minimum (%)	Maximum (%)
Stress	$k_{\sigma } = 0.05/T$	80kPa	320kPa	44kPa	291kPa	– 45	– 9
	$k_{\sigma }=0.15/T$	80kPa	120kPa	95kPa	105kPa	19	– 12
Collagen	$k_{\sigma }=0.05/T$	0.8	6.1	0.73	5.03	– 9	– 18
	$k_{\sigma }=0.15/T$	1.0	1.6	1.00	1.44	0	– 10

Gain-parameter $k_{\sigma }=k^m_{\sigma }=k^{c_i}_{\sigma }$ and turnover time $T=T^m=T^{c_i}$ from the equation of rate mass degradation and deposition (details in supplemental materials, section C)

### Applications

Our new model was then applied to simulate G&R in different types of ATA geometries. For each case, we predicted temporal evolutions of the lumen diameter and of the wall thickness, as defined in Fig. 2, following an initial insult. The material properties and initial densities were assigned as described in supplemental materials, section D.

#### Initial insult for G&R

The G&R in the arterial model is triggered by either an initial insult consisting in a sharp spatial-temporal degradation of elastin (Eq. 13) or by the half-life degradation of elastin (Eq. 14), such as,

13 $$\begin{aligned}&\varrho _R^l(t) = \varrho _{R0}^l\exp \left( \frac{-t}{T^l}\right) \nonumber \\&+ \varrho _{R0}^l\frac{D_{\max }}{t_{\text {dam}}}\exp \left( -0.5\left[ \frac{X_d}{L_{\text {dam}}}\right] ^2\right) \frac{t_{\text {dam}} T^l}{t_{\text {dam}}-T^l}\left[ \exp \left( \frac{-t}{T^l}\right) - \exp \left( \frac{-t}{t_{\text {dam}}}\right) \right] \end{aligned}$$

or

14 $$\begin{aligned} \varrho _R^l(t) = \varrho _{R0}^l\exp \left( \frac{-t}{T^l}\right) , \end{aligned}$$

where $D_{\max }$=0.5, $L_{\text {dam}}$=10-mm, and $t_{\text {dam}}$=40-days are parameters related to elastin evolution, $T^l$ is the half-life time of elastin, $\varrho ^l_R$ and $\varrho ^l_{R0}$ are the time and the initial elastin densities (per unit reference volume), respectively, and where the degradation in Eq. 13 is based on the distance $X_d$ between a given position ($\mathbf {P}_x$) and the central degradation position ($\mathbf {P}_0$), according to

15 $$\begin{aligned} X_d = \left\{ \begin{array}{ll} || \mathbf {P}_x - \mathbf {P}_0 || &{} \text {in the radial direction}, \\ &{} \\ (\mathbf {P}_x - \mathbf {P}_0) \cdot \mathbf {v} &{} \text {in the axial direction}, \\ \end{array} \right. \end{aligned}$$

#### Idealized ATA model

An idealized bi-layer model of an ATA, similar to the ones in Alford and Taber (2008) and Mousavi et al. (2019), was defined as a first test case for the sake of simplicity, given the symmetries. The geometry was an eighth of a torus ($\phi =\pi /2$) of diameter *d*=36mm and arch radius *R*=65mm, Fig. 2. Radial rollers with springs were used to ensure appropriate boundary conditions at both ends, as shown in Fig. 2. The pressure, mechanical parameters and densities are reported in Table  3 with $\beta =40.$

**Table 3:** Material parameters used in our models to simulate G&R in an idealized toric aortic arch and in a patient-specific ATA geometry

	Symbol	Value
*Geometry and load*		
Thickness	*t*	2.38mm
Pressure	*p*	80mmHg
*Mechanical properties*		
Neo-Hookean	$\mu ^l$	80J/kg
Bulk modulus	$\kappa ^l$	$\beta \times$ 80J/kg
Fung-quadratic, collagen	$k_1^{c_i}$	292.0J/kg
	$k_2^{c_i}$	5.6
Passive, SMC	$k_1^m$	13.8J/kg
	$k_2^m$	6.0
*Density media*		
Elastin	$\varrho _{R0}^l$	169.0kg/m$^3$
SMC	$\varrho _{R0}^m$	735.0kg/m$^3$
Collagen (0 and $\pi /2$)	$\varrho _{R0}^{c_1}=\varrho _{R0}^{c_4}$	14.6kg/m$^3$
Collagen ($-\pi /4$ and $\pi /4$)	$\varrho _{R0}^{c_2}=\varrho _{R0}^{c_3}$	58.4kg/m$^3$
*Density adventitia*		
Elastin	$\varrho _{R0}^l$	565.0kg/m$^3$
SMC	$\varrho _{R0}^m$	0.0kg/m$^3$
Collagen (0 and $\pi /2$)	$\varrho _{R0}^{c_1}=\varrho _{R0}^{c_4}$	48.5kg/m$^3$
Collagen ($-\pi /4$ and $\pi /4$)	$\varrho _{R0}^{c_2}=\varrho _{R0}^{c_3}$	194.0kg/m$^3$
*Initial remodeling (prestretch)*		
SMC	$\lambda _r^m(t=0)$	$1.1^{-1}$
Collagen	$\lambda _r^{c_i}(t=0)$	$1.1^{-1}$
Turnover period		
Collagen and SMC	$T^{c_i}=T^m$	101days
Elastin	$T^l$	101years

The parameters are introduced with their respective models in the supplemental material, section B

#### Patient-specific ATA model

A patient-specific geometry of an ATA was reconstructed from the CT scan of a patient harboring an aneurysm (Mousavi et al. 2019), as shown in Fig. 2. Two layers were defined across the thickness, namely the media and the adventitia. The boundary conditions at the proximal and distal ends were the same as for the idealized ATA, with radial rollers and springs, Fig. 2. The pressure, mechanical parameters and densities are reported in Table 3, with $\beta =20$.

Moreover, we also tested that our computational framework is capable to keep the homeostatic condition when there is no elastin degradation. The test was performed on the patient-specific geometry over a duration of 6000 days. The change of diameter was lower than 0.02%.

## Results

### Idealized ATA model

An initial prestretch was assigned for the elastin-matrix component as shown in Fig. 2, with $\lambda ^l_{h\Phi -IC}=0.75$, $\lambda ^l_{h\Phi -OC}=1.3$ and $\lambda ^l_{h\Theta -IC}=1.45$, $\lambda ^l_{h\Theta -OC}=1.3$. The initial prestretch followed a linear distribution between the inner and the outer curvature. This permitted to reduce distortions below 2% in the diameter and 3% in the thickness, respect to the metrics shown in Fig. 2. The stiffness of elastic boundaries, which also contribute significantly to reduce distortions, were set to 1-Pa/m and 0.2-Pa/m at the proximal and distal ends, respectively. These initial conditions eventually permitted to achieve G&R simulations for more than 10-years in this idealized ATA geometry.

**Fig. 5:**
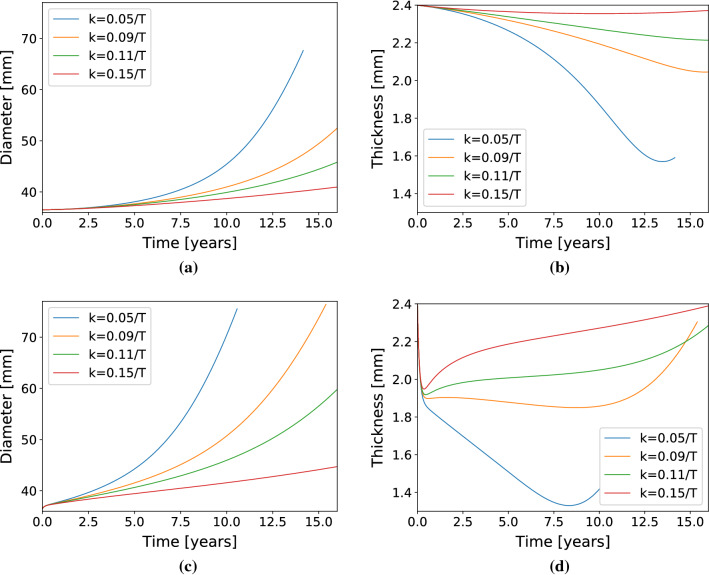
Diameter (**a**) and thickness (**b**) evolution in the idealized ATA geometry in response to half-life elastin degradation. Diameter (**c**) and thickness (**d**) evolution of the idealized ATA in response to an initial insult (sharp elastin degradation).

In Fig. 5a and c, we show the influence of the gain-parameter on the rate of aneurysm growth obtained with this model. The lower the gain parameter values, the faster the diameter changes. Such parameter has also an influence on the thickness evolutions, as shown in Fig. 5b and d. The lower the gain parameter values, the faster the thickness decreases. When the gain-parameter values become too low, the G&R becomes unstable. In that case, the lumen diameter grows above 65-mm or 70-mm. The effect is amplified when G&R follows a localized lesion, as shown in Fig. 5c).

**Fig. 6:**
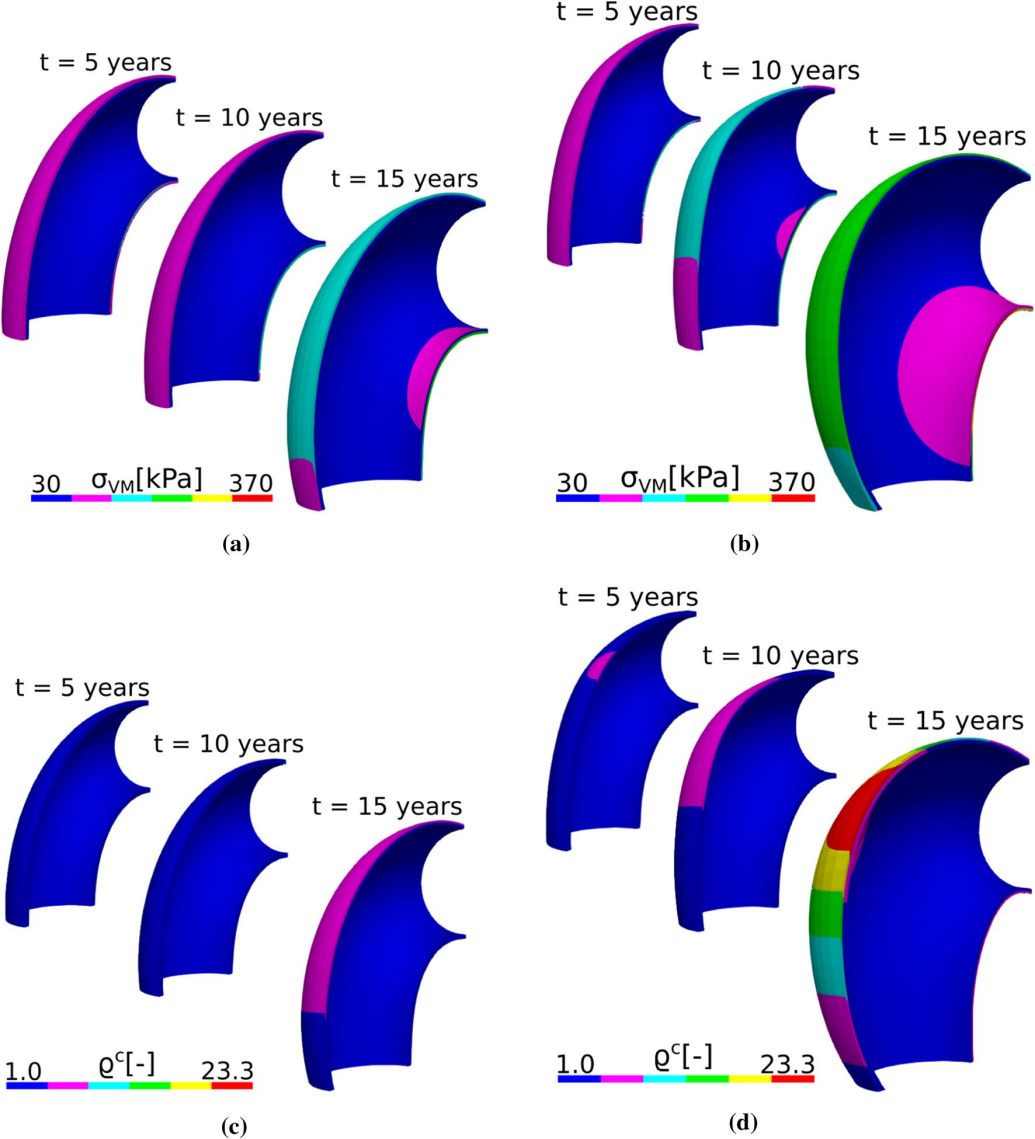
Von Mises stress ($\sigma _{VM}$) evolution in response to half-life elastin degradation (**a**) and in response to an initial insult (sharp elastin degradation) (**b**) for the idealized ATA geometry. Normalized total collagen density ($\rho ^c$) evolution in response to long-term elastin degradation (**c**) and localized elastin loss (**d**) for the idealized ATA geometry. Simulations were achieved with $k_{\sigma }=0.09/T$

Figure 6a and b show the von Mises stress field for the two cases of elastin degradation (aging and insult, respectively). The stresses are higher for the localized elastin loss than for the long-term elastin degradation. There is still a similar stress distribution for both cases, as the larger stresses are in the IC of the arch and in the adventitia while the media does not exhibit major changes of stress distribution. The story is slightly different for the total collagen distribution, where the largest production of collagen occurs at the OC of the arch and in the adventitia, as shown in Fig. 6c and d.

### Patient-specific ATA model

The initial prestretch of elastin was distributed with a linear gradient as shown in Fig. 2 with $\lambda ^l_{h\Phi -IC}=0.75$, $\lambda ^l_{h\Phi -OC}=1.3$ and $\lambda ^l_{h\Theta -IC}=1.5$, $\lambda ^l_{h\Theta -OC}=1.0$. Distortions lower than 6% were obtained in the diameter metric and below 3% in the thickness at IC and OC—the distortions are measured respect to the metrics shown in Fig. 2—. We allowed more distortion in the homeostatic diameter as a trade-off between distortions and stability. Indeed, reducing initial distortions implied applying larger pre-tensions in the initial homeostatic state. Such large pre-tensions were systematically responsible for further instability in the G&R simulations. For instance, average stress values above 200 kPa for the homeostatic state were systematically responsible for a divergence of the FE analysis after 100 days of G&R. Spring stiffness values of 10 Pa/m and 0.5 Pa/m were assigned at the proximal and distal boundaries, respectively.

**Fig. 7:**
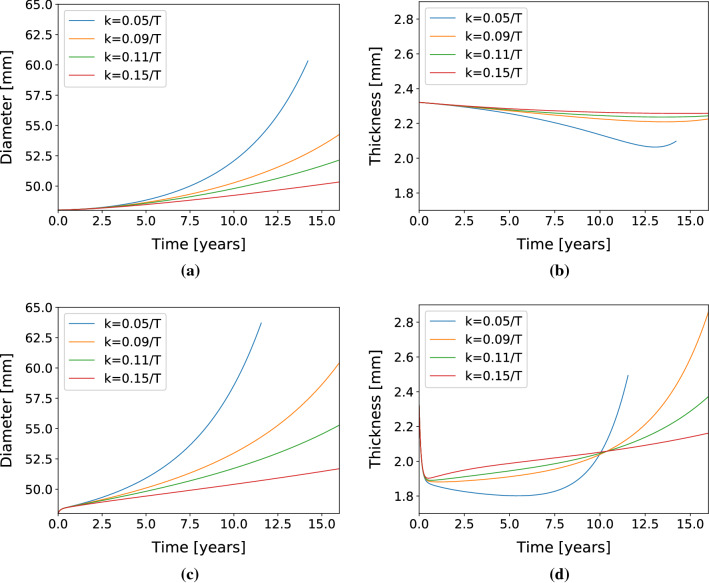
Diameter (**a**) and thickness (**b**) evolution in the patient-specific ATA geometry in response to half-life elastin degradation. Diameter (**c**) and thickness (**d**) evolution of the patient-specific ATA in response to an initial insult (sharp elastin degradation)

As the patient already harbored an ATA aneurysm, the reference homeostatic lumen diameter was 48 mm. Further diameter evolutions predicted by the model are shown in Fig. 7a and c. As for the idealized ATA, these evolutions depend on the gain parameter and on the type of insult (acute or long-term elastin loss). When the gain-parameter values become too low, the G&R becomes unstable after 60 mm diameter. Differences with respect to the idealized case are visible in the thickness evolution, as shown in Fig. 7b and d. For long-term elastin loss, the thickness decreases more slowly than with the acute insult, the latter showing a sharp decrease in the first year due to the acute elastin loss.

**Fig. 8:**
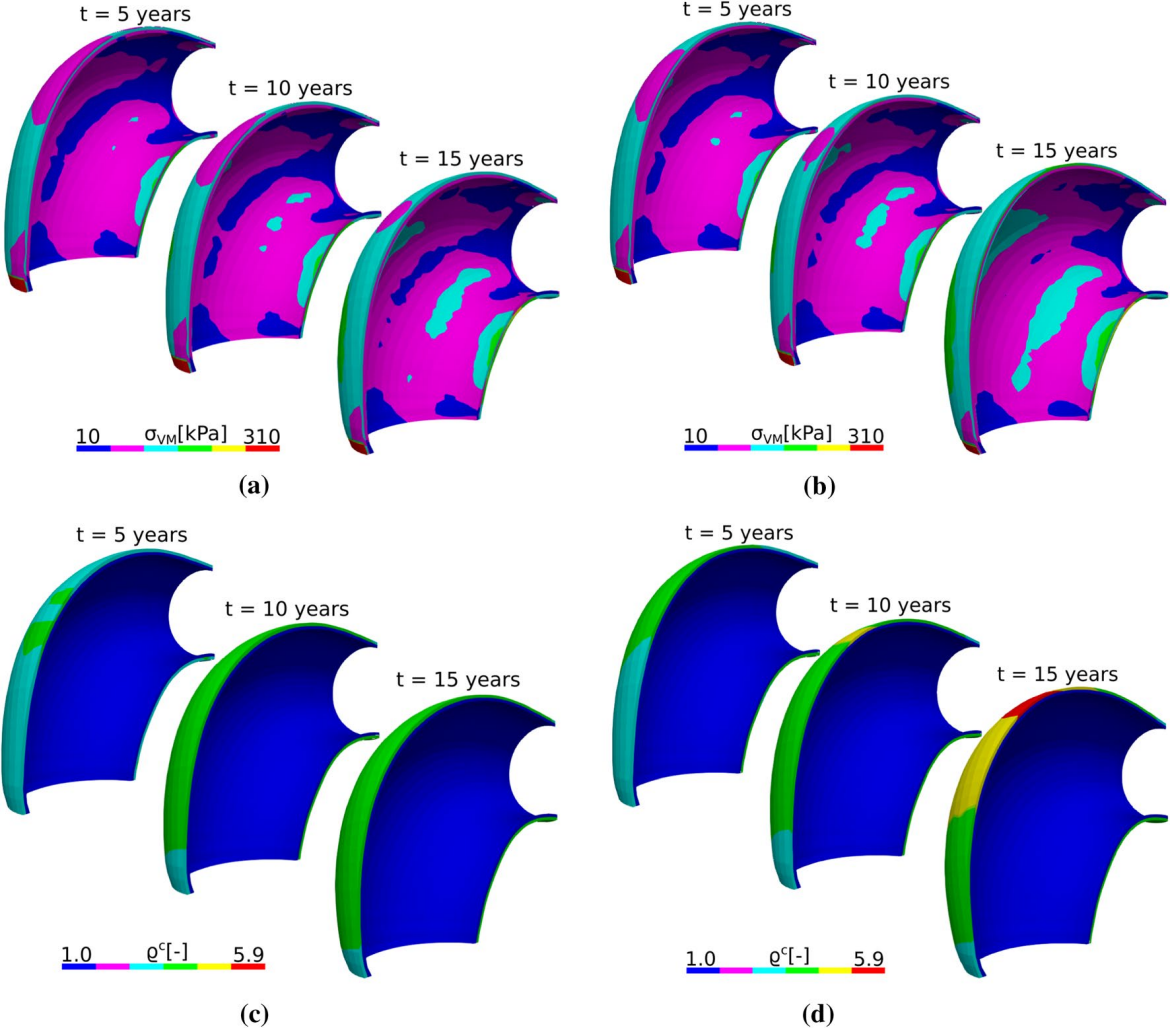
Von Mises stress ($\sigma _{VM}$) evolution in response to half-life elastin degradation (**a**) and in response to an initial insult (sharp elastin degradation) (**b**) for the patient-specific ATA geometry. Normalized total collagen density ($\rho ^c$) evolution in response to long-term elastin degradation (**c**) and localized elastin loss (**d**) for the patient-specific ATA geometry. Simulations were achieved with $k=0.09/T$

The stress distribution for the patient-specific ATA model shows a pattern of high stresses in the axial direction in both layers (media and adventitia). The largest stress values are found in the adventitia for both cases, as shown in Fig. 8a and b. A very large stress concentration is obtained in the adventitia at the OC near the proximal edge. Despite the stress pattern and stress concentrations, collagen accumulates mainly in the adventitia at the OC and far from the boundaries for the long-term elastin loss, whereas collagen concentration is more pronounced in the elastin degradation zone for the acute insult, as shown in Fig. 8c and d.

## Discussion

Mechanoregulation of collagen tension in soft tissues is essential in the progression of aortic aneurysms. In this work, we established a numerical model to simulate these effects in the thoracic aorta. The model is based on the constrained mixture theory, which requires that prestretches are assigned to each microstructural component of the arterial tissue. Although these prestretches actually exist even in the initial reference configuration, they have never been measured accurately and we need to make assumptions in order to assign them in computational models. They were previously assumed as homogeneous in idealized straight tubes, but in the present study, we investigated more elaborate prestretch distributions for curved arteries, with a special focus on a patient-specific ATA geometry.

We proposed to assign a prestretch gradient from the inner to the outer ATA curvature. This permitted to establish a compatible initial homeostatic state and to produce stable and long (above 10-years) simulations using our 3D FE implementation of G&R. This gradient was needed to compensate the different effects of the lumen pressure at the IC and OC in a curved vessel (Fig. 2). This non-uniform prestretch is in agreement with the previous study of Alford and Taber (2008), who already considered a different axial prestretch at the IC and at the OC of an idealized toric aortic arch. Outside this study, the homeostatic deposition stretch of elastin was usually assigned as uniform in previously CM models dedicated to straight geometries (Valentín et al. 2013; Lin et al. 2017; Horvat et al. 2019). Although a linear distribution of longitudinal and circumferential prestretches gives stability to G&R simulations, this remains a simplification for the sake of computational analyses. The current work suggests for the first time the existence of these spatial variations of prestretches in ATAs. However, the spatial distribution of prestretches could be more complex. For example, other distributions based on polynomial expressions could be defined to ATAs and optimized through sensitivity analyses (Brandstaeter et al. 2021). Furthermore, the linear assumption has not been experimentally validated. Experimental investigations could consist in measuring the shortening of ATAs in the inner and outer curvature after excision. Nevertheless, Alford and Taber (2008) highlighted the difficulties to measure residual stresses. Therefore, the actual distribution of prestretches remains an open problem.

In addition to initial prestretches, other factors appeared to be critical to perform stable G&R simulations in curved arterial geometries. One of these factors is the boundary condition at the proximal and distal borders. We used radial rollers, as shown in Fig. 2, and as detailed in supplemental materials, section A. These boundary conditions are consistent with the actual conditions of an ATA, which is tethered elastically in the body, for instance to the heart. The elastic radial rollers also helped to reduce stress concentration at the proximal border in the patient-specific ATA simulations. Similar boundary conditions were previously used by Nama et al. (2020) and Moireau et al. (2012) to model the external tissue support of the aorta (e.g. the surrounding organs), but our study is the first one dedicated to G&R.

Although we ensured that mechanical equilibrium was satisfied in the reference homeostatic configuration, our choice of the longitudinal prestretch remained arbitrary (Mousavi et al. 2019), as it has never been characterized in human ATA. Even the linear gradient of prestretch in the radial direction and the parameters of Robin boundary conditions were chosen mostly for the sake of keeping stability in the simulations. There are always many constitutive parameters in G&R models, and more experimental data—especially on humans—are still necessary to set the parameter values more accurately.

Different models were previously used for the elastin in G&R simulations based on the CM model. We used a three-dimensional neo-Hookean nearly incompressible model, as detailed in supplemental materials, section B. This is a major difference with the model proposed by Braeu et al. (2017), where a combination of two- and three-dimensional formulations was implemented to ensure numerical stability. Although our model omitted the 2D part, we obtained still a very good agreement with their results in the verification case.

Our model captured aneurysm progression in the ATA, either with an idealized toric geometry or in a patient-specific geometry. For low gain parameters ($k^j_{\sigma }<0.1/T^j$), the diameter reached 55 mm (Figs. 5 and 7), which is the threshold at which surgical repair is recommended, along with growing rates above 1.5 mm/year (Trabelsi et al. 2015). However, the respective von Mises stress values shown in Figs. 6b and 8b remained below 500 kPa (Trabelsi et al. 2015; Duprey et al. 2016; He et al. 2021). Moreover, high gain parameters ($k^j_{\sigma }>0.1/T^j$) induced less pronounced dilatations, with dimeters remaining below the 55 mm threshold and stabilization of aneurysm evolution.

In the patient-specific simulation modeling the aging effect, the gain parameters $k^j_{\sigma }=\{0.11,0.15\}/T^j$ produced dilatation of 1.2-mm(3.7%) and 1.8-mm(2.5%) in the first decade, respectively. Such ATA diameter increases are in good agreement with previous observations made by Redheuil et al. (2011) and Sugawara et al. (2008), with progressions of 1.1-mm and 3% per decade, respectively. Aging is related with elastin fragmentation and possible fibrosis causing stiffening of the arterial wall (Vlachopoulos et al. 2011; O’Rourke et al. 2008). Our aging simulations captured well such effects by taking into account the half-life time of elastin in Eq. 14. Elastin degradation induced an accumulation of collagen, especially in the adventitia, which could be responsible for fibrosis and ATA stiffening. However, we did not consider possible pressure increase due to hypertension that can be indirectly caused by the increased stiffness (Redheuil et al. 2011; Vlachopoulos et al. 2011). Other effects remained neglected in our work, such as aortic unfolding, which is the natural increase of radius of curvature of the aortic arch with age (Redheuil et al. 2011). Including this effect in the model would require updating the boundary conditions with time, especially at the aortic root. There is still a lack of knowledge about these effects and they should be investigated further in the future.

In the definition of the prestretch, we assumed a uniform wall thickness in the reference configuration (Mousavi et al. 2019). Although the thickness of the aorta is not uniform, it is not possible to measure it accurately with currently available in vivo imaging modalities. Zeinali-Davarani et al. (2011a) proposed a methodology for optimizing the thickness in the definition of the homeostatic state. Such optimization could be considered in a future work.

## Conclusions

In this work, we implemented the homogenized constrained mixture model in a three-dimensional FE framework and we used this framework to simulate aneurysm development and progression in a patient-specific ATA, assuming that G&R works at maintaining a homeostatic level of tension in collagen fibers. We especially modelled aneurysm progression following localized elastin degradation in the ATA. As ATAs are curved arteries, heterogeneous initial prestretches had to be initially assigned to the constituents of the constrained mixture in order to satisfy equilibrium and set initial homeostatic conditions preceding the initial insult. It was critical to initiate G&R simulations with homeostatic conditions but these conditions also determine further aneurysm progression. This work highlighted the complexity of prestretches in FE models simulating G&R of the ATA based on tensional homeostasis. Such prestretches should actually reflect all the developmental history of each individual and future work will focus on identifying these prestretches in vivo.

## Supplementary Information

Below is the link to the electronic supplementary material.

Supplementary material 1 (pdf 139 KB)

## Data Availability

https://github.com/jdlaubrie/Kuru.

## Code availability

https://github.com/jdlaubrie/Kuru.
