# Supplementary material for: About prestretch in homogenized constrained mixture models simulating growth and remodeling in patient-specific aortic geometries
Source: Biomech Model Mechanobiol. 2022 Jan 24;21(2):455–69. doi: 10.1007/s10237-021-01544-3 (PMC8940846; doi:10.1007/s10237-021-01544-3)
Supplement: Supplementary file 1 — Supplementary material 1 (pdf 139 KB) [file 10237_2021_1544_MOESM1_ESM.pdf]

Biomechanics and Modeling in Mechanobiology manuscript No.
(will be inserted by the editor)

About prestretch in homogenized constrained mixture models: simulating growth and remodeling in patient-specific aortic geometries

Joan D. Laubrie · S. Jamaleddin Mousavi · Stéphane Avril

Received: date / Accepted: date

A Theoretical framework

Let us consider a material mixture composed by several constituents ($j = 1,2,...,n$) in its hypothetical traction-free reference configuration $\boldsymbol{\Omega}_R \subset \mathbb{R}^3$ and subsequently deformed into a loaded configuration $\boldsymbol{\Omega}_t \subset \mathbb{R}^3$ at time $t \geq 0$ (Fig. 1). The deformation map $\chi : \mathbb{R}^+ \times \boldsymbol{\Omega}_R \rightarrow \mathbb{R}^3$ which maps a material point $\boldsymbol{X} \in \boldsymbol{\Omega}_R$ to the corresponding spatial point $\boldsymbol{x} = \chi(\boldsymbol{X}, t) \in \boldsymbol{\Omega}_t$ produces a deformation gradient of the mixture defined as

$$\boldsymbol{F} = \frac{\partial \boldsymbol{x}}{\partial \boldsymbol{X}}, \tag{S.1}$$

where this total deformation incorporate an elastic and inelastic deformation for each jth constituent in the mixture, equation 1.
It is assumed that the material mixture is hyperelastic and depends only on the elastic deformation of each constituent, *i.e.* on the elastic Cauchy-Green tensor

$$\boldsymbol{C}_e^j = [\boldsymbol{F}_{gr}^j]^{-T} \boldsymbol{C} [\boldsymbol{F}_{gr}^j]^{-1}, \tag{S.2}$$

where $\boldsymbol{C} = \boldsymbol{F}^T \boldsymbol{F}$ is the Cauchy-Green tensor related to the deformation gradient defined in Eq. 1. Thus, from the equations 3 and S.2 is possible to define stress in the jth constituent as,

$$\boldsymbol{S}^j = 2 \frac{\rho_R^j}{\phi^j} \frac{\partial \Psi^j}{\partial \boldsymbol{C}} \tag{S.3}$$

where the sum of the all individual stresses give the second Piola-Kirchhoff stress of the whole mixture ($\sum_j^n \phi^j \boldsymbol{S}^j$), with the constituent mass fraction $\phi^j = \frac{\rho_R^j}{\rho_R}$ and the density of the mixture ($\rho_R = \sum_j^n \rho_R^j$). The $\boldsymbol{S}^j$

Joan D. Laubrie
Mines Saint-Étienne, Univ Lyon, Univ Jean Monnet, INSERM, U 1059 Sainbiose, F - 42023 Saint-Étienne France
ORCID: 0000-0002-2423-9919

S. Jamaleddin Mousavi
Mines Saint-Étienne, Univ Lyon, Univ Jean Monnet, INSERM, U 1059 Sainbiose, F - 42023 Saint-Étienne France
ORCID: 0000-0003-0509-1450

Stéphane Avril
Mines Saint-Étienne, Univ Lyon, Univ Jean Monnet, INSERM, U 1059 Sainbiose, F - 42023 Saint-Étienne France
ORCID: 0000-0002-8604-7736
E-mail: avril@emse.fr

can be mapped to the spatial configuration by the push-forward transformation

$$\boldsymbol{\sigma}^j = \frac{1}{J}\boldsymbol{F}\boldsymbol{S}^j\boldsymbol{F}^T \tag{S.4}$$

with $J = \det\boldsymbol{F}$.
During G&R the tissue is continously adapting and consequently is changing its structure with deposition/removal of mass of the constituents, for instance, elastin degradation, SMCs apoptosis/proliferation or collagen production by fibroblasts/SMCs. The mass changes in the mixture can be written like,

$$\dot{m} = \frac{D}{Dt}(\rho_R V) = \frac{D}{Dt}(\rho v), \tag{S.5}$$

where $\dot{m}$ is the depositon/removal of mass in the mixture, $\rho_R V$ is the density times the volume in reference configuration, ρv is the density times the volume in spatial configuration. The reference volume V does nont change, the spatial density is constant in time and homogeneous, and with the relation between the volumes $v = JV$ ($\rho_R = \rho J$), we can write the mass changes in reference and spatial configurations as it follows Rodriguez et al. (1994),

$$\frac{\partial \rho_R}{\partial t} = J\rho \operatorname{div}\boldsymbol{v}, \tag{S.6}$$

then, the reference density (per unit reference volume) change over time $\rho_R = \rho_R(t)$ Cyron et al. (2016); Braeu et al. (2017). Furthermore, G&R occurs at slow time scales, then it can be assumed that it is quasi-static and the linear momentum balance equals zero,

$$\frac{D}{Dt}(\rho\boldsymbol{v}) = \operatorname{div}\boldsymbol{\sigma} + \rho\boldsymbol{b} = 0 \tag{S.7}$$

where $\boldsymbol{v}$ is the velocity of the system, $div\boldsymbol{\sigma}$ is the diveregence of the mixture Cauchy stress $\boldsymbol{\sigma}$ and the body force $\boldsymbol{b}$, in the spatial configuration.
At the surface of the body the conditions can be, given deformations (Dirichlet), external loads applied on the body surface (Neumann) or deformation-dependent forces (Robin, Moireau et al. (2012); Nama et al. (2020)). The Robin boundary conditions are introduced by the following expression:

$$\boldsymbol{\sigma}\cdot\boldsymbol{n} = p\boldsymbol{n} + k\boldsymbol{u}, \tag{S.8}$$

where p denotes the pressure, $\boldsymbol{n}$ the normal to the surface, the elastic forces with stiffness k and displacement $\boldsymbol{u}$. This forces are appropriate for the modelization of the lumen pressure in blood vessels due to the blood flow, and the elastic forces are usefull to give flexible and stable boundary displacements.

B Constitutive Models

At the equation 2 is assumed that the mixture strain energy W per unit reference volume is the sum of the constituents strain energies Ψ^j per unit mass. Therefore, we consider our material to be vascular tissue composed by three constituents such as, elastin, SMCs and collagen. The elastin is assumed to be isotropic; the smooth muscle have an active, $(\bullet)_{act}$, and a passive ,$(\bullet)_{pas}$, behavior; and the collagen is composed by four fibre families aligned in circumferential, longitudinal and two diagonal directions, respectively. The total strain energy may be written such as

$$W = \rho_R^l\Psi^l + \rho_R^m(\Psi_{pas}^m + \Psi_{act}^m) + \sum_{i=1}^{4}\rho_R^{c_i}\Psi^{c_i}, \tag{S.9}$$

for each component we assumed a strain energy to represent the corresponding hyperelastic behavior. We modeled the elastin with a Neo-Hookean hyperelastic model as in Mousavi and Avril (2017); Mousavi et al. (2018)

$$\Psi^l = \frac{\mu^l}{2}\left(\boldsymbol{I}:\bar{\boldsymbol{C}}_e^l - 3\right) + \frac{\kappa^l}{2}\left(J_e^l - 1\right)^2 \tag{S.10}$$

where μ^l and κ^l are stress-like material parameters (shear and bulk modulus), while $\bar{\boldsymbol{C}}_e^l$ and J_e^l are the elastic isochoric right Cauchy-Green tensor and the elastic jacobian of elastin, respectively. The isochoric right Cauchy-Green tensor is related to the right Cauchy-Green tensor by $\bar{\boldsymbol{C}}_e^l = (J_e^l)^{2/3}\boldsymbol{C}_e^l$ and the elastin elastic jacobian is $J_e^l = \det(\boldsymbol{F}_e^l)$.

We modeled the collagen fibre families by an anisotropic Fung-type exponential function such as

$$\Psi^{c_i} = \frac{k_1^{c_i}}{2k_2^{c_i}}\left(\exp(k_2^{c_i}(\lambda_e^{c_i 2}-1)^2)-1\right) \tag{S.11}$$

where $k_1^{c_i}$ and $k_2^{c_i}$ are a stress–like and dimensionless material parameters, respectively, while $\lambda_e^{c_i}$ is the elastic stretch contribution of the collagen fibre obtained as

$$\lambda_e^{c_i} = \frac{\lambda^{c_i}}{\lambda_r^{c_i}}\ \text{with}\ \lambda^{c_i} = \sqrt{\boldsymbol{C}:(\boldsymbol{a}_0^{c_i}\otimes\boldsymbol{a}_0^{c_i})}\ \text{and}\ \lambda_r^{c_i} = \|\boldsymbol{F}_{gr}^{c_i}\cdot\boldsymbol{a}_0^{c_i}\| \tag{S.12}$$

where λ^{c_i} and $\lambda_r^{c_i}$ are the total and remodeling stretch (cf. 1) of the fibre, respectively. We also modeled the passive behavior of SMCs by an anisotropic Fung-type exponential function such as

$$\Psi_{pas}^{m} = \frac{k_1^{m}}{2k_2^{m}}\left(\exp(k_2^{m}(\lambda_e^{m 2}-1)^2)-1\right) \tag{S.13}$$

where k_1^m and k_2^m are stress–like and dimensionless material parameters, respectively, λ_e^m is the elastic contribution of SMCs calculated such as

$$\lambda_e^{m} = \frac{\lambda^{m}}{\lambda_r^{m}}\ \text{with}\ \lambda^{m} = \sqrt{\boldsymbol{C}:(\boldsymbol{a}_0^{m}\otimes\boldsymbol{a}_0^{m})}\ \text{and}\ \lambda_r^{m} = \|\boldsymbol{F}_{gr}^{m}\cdot\boldsymbol{a}_0^{m}\| \tag{S.14}$$

where λ^m and λ_r^m are the total and remodeling stretch (cf. 1) of the fibre, respectively. While we modeled its active behavior according to Braeu et al Braeu et al. (2017),

$$\Psi_{act}^{m} = \frac{\sigma_{actmax}}{\rho_{R0}}\left(\lambda_{act} + \frac{(\lambda_{max}^{m}-\lambda_{act})^3}{3(\lambda_{max}^{m}-\lambda_0^{m})^2}\right) \tag{S.15}$$

with σ_{actmax} the maximal active Cauchy stress, λ_{act} is the active stretch in the fibre direction, λ_0^m and λ_{max}^m are the zero and maximum active stretches and ρ_{R0} denotes the total mixture density in the homeostatic reference configuration.

C Growth and Remodeling

The idea of the hCM models is to pool all the sequential mass additions within one single change using temporal homogenization (Fig. 1). To do so, three assumptions are made: (i) the mechanical properties are changed by G&R, (ii) survival mass (mass turnover) functions are exponential and (iii) inelastic deformations, $\boldsymbol{F}_{gr}^j = \boldsymbol{F}_g^j\boldsymbol{F}_r^j$, are in turn decomposed into growth-related, $\boldsymbol{F}_g^j$, and remodeling-related (turnover-related), $\boldsymbol{F}_r^j$, contributions. In this model a single local average inelastic deformation gradient $\boldsymbol{F}_{gr}^j$ is defined by constituent. The model can handle isotropic or anisotropic growth, the latter being more relevant for arteries and manifesting with thickening or thinning effects Matsumoto and Hayashi (1996).
We assumed that G&R is a stress mediated process which tends to minimize deviations between the current stress and a reference stress metrics named homeostatic stress ($\boldsymbol{\sigma}_h$). Therefore, the rate of mass degradation and deposition at time t for the jth constituent is expressed as

$$\dot{\rho}_R^j = \rho_R^j k_\sigma^j \frac{\sigma^j - \sigma_h^j}{\sigma_h^j} + \dot{D}^j \tag{S.16}$$

where ρ_R^j is the mass density (per unit reference volume) of the jth constituent at time t, k_σ^j denotes a mass-gain parameter, σ^j is the spatial stress along the fibre ($\sigma^j = (\boldsymbol{a}_0^j\otimes\boldsymbol{a}_0^j):\boldsymbol{\sigma}^j$, cf S.4), $\dot{D}^j$ includes any additional mass deposition or degradation governed by non-mechanical effects (for instance the effect of a drug). Equation S.16 is related to the equation S.6 through the addition of constituents mass $\sum_j \rho_R^j = \rho_R$. A more general form of this equation is presented by Braeu et al Braeu et al. (2017), using a tensorial representation and possibly considering wall shear stress stimuli Taber (1998); Figueroa et al. (2009). The wall shear stress effects induced by the blood flow are neglected in our work here.
Braeu et al Braeu et al. (2017) assumed that growth captures local change of volume induced by mass variations of each constituent, and that all components of the mixture share the same growth deformation: $\boldsymbol{F}_g^j = \boldsymbol{F}_g$.

Then, the growth is measured from the changes of the reference mass density (ρ_R) respect to the initial ($t_0 = 0$) reference mass density (ρ_{R0}) through

$$\det(\boldsymbol{F}_g) = \frac{\rho_R}{\rho_{R0}}, \tag{S.17}$$

noting that the mixture volume changes are measured by the determinant of the deformation gradient $J = \det(\boldsymbol{F})$ and assuming the elastic and remodeling processes are isochoric ($\det(\boldsymbol{F}_e^j) = \det(\boldsymbol{F}_r^j) = 1$), so, the mixture volume changes remains equal to the growth $J = \det(\boldsymbol{F}_g)$. If the growth is assumed to be anisotropic and along the thickness direction ($\boldsymbol{a}_0^\perp$), can be expressed in tensorial form as

$$\boldsymbol{F}_g = \boldsymbol{I} + \frac{\rho_R}{\rho_{R0}} \boldsymbol{a}_0^\perp \otimes \boldsymbol{a}_0^\perp - \boldsymbol{a}_0^\perp \otimes \boldsymbol{a}_0^\perp \tag{S.18}$$

where $\boldsymbol{I}$ is the identity second order tensor. Therefore, due to the continuous mass deposition and removal, the traction-free configuration change during G&R (Fig. 1), even if there is a balance between mass deposition and removal ($\dot{\rho}_R^j = 0$), this occur with a prestress which is different from the current stress at which mass is removed or deposited. Altogether leads to changes of tissue microstructure referred as remodeling. Therefore, assuming that remodeling occurs at a constant volume and along a fibre in the direction $\boldsymbol{a}_0^j$, the evolution of the remodeling of the jth constituent at time t is expressed such as Cyron et al. (2016)

$$\dot{\lambda}_r^j = \left(\frac{\dot{\rho}_R^j}{\rho_R^j} + \frac{1}{T^j} \right) \frac{\lambda^j}{(\lambda_e^j)^2} \left(\frac{\partial \sigma^j}{\partial \lambda_e^j} \right)^{-1} \times \left(\sigma^j - \sigma_{pre}^j \right). \tag{S.19}$$

where subscript "pre" indicates prestress, $\dot{\lambda}_r^j$ denotes the remodeling velocity and T^j is the average turnover time during which old mass increment is degraded and replaced by a new mass increment. According to proposition 1 from Cyron and Humphrey Cyron and Humphrey (2016), the prestress σ_{pre}^i is equal to the homeostatic stress σ_h^i. The remodeling of the fibre can be represented in tensorial form as

$$\boldsymbol{F}_r^j = \lambda_r^j \boldsymbol{a}_0^j \otimes \boldsymbol{a}_0^j + \frac{1}{\sqrt{\lambda_r^j}} (\boldsymbol{I} - \boldsymbol{a}_0^j \otimes \boldsymbol{a}_0^j), \tag{S.20}$$

finally, from equations S.18 and S.20 is possible to get the inelastic deformation tensor or growth and remodeling for a fibre as

$$\boldsymbol{F}_{gr}^j = \lambda_r^j \boldsymbol{a}_0^j \otimes \boldsymbol{a}_0^j + \frac{1}{\sqrt{\lambda_r^j}} (\boldsymbol{I} - \boldsymbol{a}_0^j \otimes \boldsymbol{a}_0^j + \frac{\rho_R}{\rho_{R0}} \boldsymbol{a}_0^\perp \otimes \boldsymbol{a}_0^\perp - \boldsymbol{a}_0^\perp \otimes \boldsymbol{a}_0^\perp) \Longrightarrow \boldsymbol{F}_{gr}^j \cdot \boldsymbol{a}_0^j = \lambda_r^j \boldsymbol{a}_0^j. \tag{S.21}$$

To calculate the G&R deformation gradient over time we solved the system composed of Eqs. S.16, S.17 and S.19 by performing temporal integration, this is applied in a precedure to carry-out G&R simulation within a FE code, figure 1.

D Material properties

The mechanical properties of the patient-specific model are fitted from data available in the literature Mousavi et al. (2018) (bulge test). For this issue we assume that the material in the bulge have bi-tangential deformation ($\boldsymbol{F} = \mathrm{diag}[\lambda_{\theta 1}\, \lambda_{\theta 2}\, \lambda_r]$) and it is incompressible ($\det\boldsymbol{F} = 1$). The computation of the experimental measures as stretch and stress are made in base of the formulation presented in Rossi et al. (2020), from where we distribute the tangential stress σ_b within the specific-layer stresses σ_M and σ_A, media and adventitia, respectively, with α as the media thickness ratio, Fig. 2.

The definition of the constituent densities is made in base of the histological observation showed in Humphrey and Holzapfel (2012), where the thoracic aorta is composed by 35% of SMCs, 35% of elastin and 30% of collagen, according to our approach of three constituent in the arterial wall. After we place the constituents ratios in a unit square (left square in Fig. 3), and from the new constituent rectangles is possible to get their vertical and horizontal dimensions. The next step is to draw two new unit squares subjected to the media/adventitia (50%/50%) ratio; so the media gets 70% of muscle, 16.2% of elastin and 13.8% of collagen; while the adventitia gets 53.8% of elastin and 46.2% of collagen. Finally, the constituent areas (or proportion) are multiplied by the mixture density, for instance, $\rho_{R0} = 1050[kg/m^3]$, which either correspond to the whole arterial wall or to each layer, Fig. 3.

References

Braeu F, Seitz A, Aydin R, Cyron C (2017) Homogenized constrained mixture models for anisotropic volumetric growth and remodeling. Biomech Model Mechanobiol 16(3):889–906

Cyron C, Humphrey J (2016) Vascular homeostasis and the concept of mechanobiological stability. Int J Eng Sci 52:645–664

Cyron C, Aydin R, Humphrey J (2016) A homogenized constrained mixture (and mechanical analog) model for growth and remodeling of soft tissue. Biomech Model Mechanobiol 15:1389–1403

Figueroa C, Baek S, Taylor C, Humphrey J (2009) A computational framework for fluid–solid-growth modeling in cardiovascular simulations. Comput Methods Appl Mech Engrg 198(45):3583 – 3602

Humphrey J, Holzapfel G (2012) Mechanics, mechanobiology, and modeling of human abdominal aorta and aneurysms. Journal of Biomechanics 45(5):805 – 814, DOI https://doi.org/10.1016/j.jbiomech.2011.11.021, special Issue on Cardiovascular Solid Mechanics

Matsumoto T, Hayashi K (1996) Response of arterial wall to hypertension and residual stress. In: Biomechanics, pp 93–119

Moireau P, Xiao N, Astorino M, Figueroa C, Chapelle D, Taylor C, Gerbeau J (2012) External tissue support and fluid–structure simulation in blood flows. Biomechanics and Modeling in Mechanobiology 11:1 – 18, DOI https://doi.org/10.1007/s10237-011-0289-z

Mousavi J, Avril S (2017) Patient-specific stress analyses in the ascending thoracic aorta using a finite-element implementation of the constrained mixture theory. Biomech Model Mechanobiol 16:1765–1777

Mousavi S, Farzaneh S, Avril S (2018) Computational predictions of damage propagation preceding dissection of ascending thoracic aortic aneurysms. Int J Numer Method Biomed Eng 34(4):e2944

Nama N, Aguirre M, Humphrey JD, Figueroa CA (2020) A nonlinear rotation-free shell formulation with prestressing for vascular biomechanics. Scientific Reports 10(1):17528, DOI https://doi.org/10.1038/s41598-020-74277-5

Rodriguez E, Hoger A, McCulloch A (1994) Stress-dependent finite growth in soft elastic tissues. J Biomech 27:455–467

Rossi F, Fumagalli F, Ruiz-Moreno A, Moilanen P, Hähner P (2020) Membrane bulge test rig for irradiation-assisted stress-corrosion cracking. Nuclear Instruments and Methods in Physics Research Section B: Beam Interactions with Materials and Atoms 479:80 – 92, DOI https://doi.org/10.1016/j.nimb.2020.06.012

Taber L (1998) A model for aortic growth based on fluid shear and fiber stresses. J Biomech Eng 120(3):348–354

List of Figures

1 FE algorithm of hCM model 6
2 Mechanical parameters 6
3 Density diagrams 7

⋆ Initialize state variables
  $\rho_R^j(0)$ and $\boldsymbol{F}_{gr}^j(0)$
⋆ Set boundary conditions
⋆ Assemble the internal stiffness matrix $\mathbf{K}_{int}$
⋆ Let the time flow n years
- If $t \neq 0$. Update state variables (G&R) and internal stiffness matrix $\mathbf{K}_{int}$
  $\rho_R^j(t+1) = \rho_R^j(t) + \dot{\rho}_R^j \Delta t$ and $\boldsymbol{F}_{gr}^j(t+1) = \boldsymbol{F}_{gr}^j(t) + \dot{\boldsymbol{F}}_{gr}^j \Delta t$
  $\dot{\rho}_R^j = \dot{\rho}_R^j(\sigma^j, \sigma_h^j)$ and $\dot{\boldsymbol{F}}_{gr}^j = \dot{\boldsymbol{F}}_{gr}^j(\boldsymbol{F}, \boldsymbol{F}_e^j, \sigma^j, \sigma_h^j)$
- Update external forces $\mathbf{F}_{ext}$ and residual $\mathbf{R}$
  $\mathbf{R} = -\mathbf{F}_{ext}$
- Newton-Raphson loop, until $\mathbf{R} \approx 0$
  ∗ Solve the equilibrium system, if first iteration $\mathbf{K} = \mathbf{K}_{int}$
    $\mathbf{K} \cdot \boldsymbol{\Delta} = \mathbf{R}$
  ∗ Compute current coordinates
    $\boldsymbol{x}(t+1) = \boldsymbol{x}(t) + \boldsymbol{\Delta}$
  ∗ Compute internal stiffness matrix $\mathbf{K}_{int}$ and internal forces $\mathbf{T}_{int}$
  ∗ Compute external stiffness matrix $\mathbf{K}_{ext}$ and external forces $\mathbf{F}_{ext}$
  ∗ Update stiffness matrix $\mathbf{K}$ and residual $\mathbf{R}$
    $\mathbf{K} = \mathbf{K}_{int} + \mathbf{K}_{ext}$ and $\mathbf{R} = \mathbf{T}_{int} - \mathbf{F}_{ext}$
- Update total displacements
  $\boldsymbol{\delta}(t+1) = \boldsymbol{x}(t+1) - \boldsymbol{X}$
- Compute CMM measures
  $\sigma^j(t+1) = \boldsymbol{\sigma}^j : (\boldsymbol{a}_0^j \otimes \boldsymbol{a}_0^j)$
- If $t = 0$. Stock constituent measures. $t = 0$ is the homeostatic step
  $\sigma_h^j = \sigma^j(t=0)$

Fig. 1 Implemented algorithm of the hCM model with forward Euler integration. Time $t = 0$ is the homestatic step and defines its constituent stress metrics for the G&R evolution.

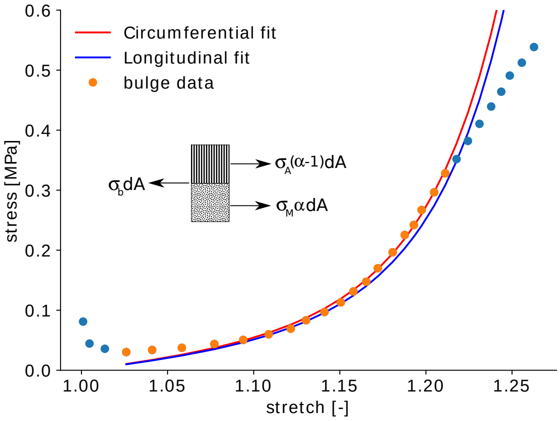

Fig. 2 Patient axial and circumferential stress-stretch curves fitted from data available in the literature Mousavi et al. (2018) (bulge test). The tangential stress (σ_b) distributed within the layers, with σ_M and σ_A the media and adventitia stress, respectively, and α is the media thickness ratio in the total arterial wall.

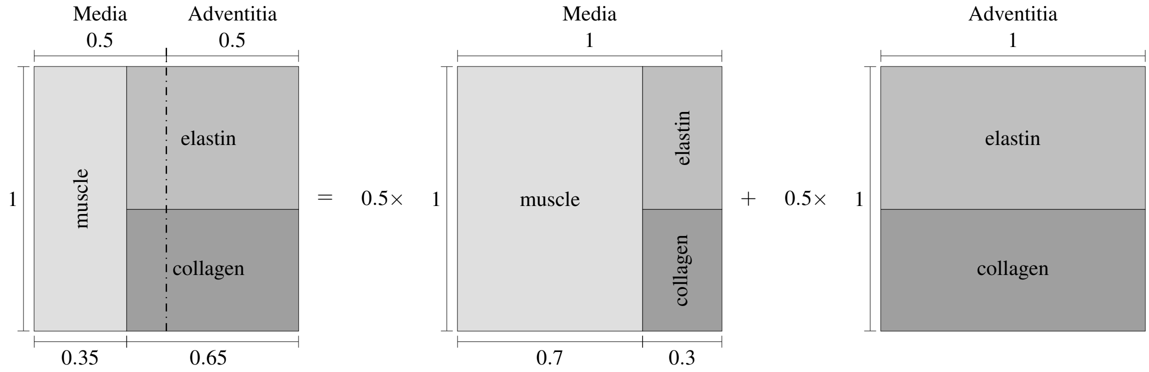

Fig. 3 The first unit square at the left represents the whole arterial wall divided in the mixture constituents, which is split into two new unit squares according to the media/adventitia ratio, each one with its corresponding mixture constituents.
